# Who Wants to Grant Robots Rights?

**DOI:** 10.3389/frobt.2021.781985

**Published:** 2022-01-13

**Authors:** Maartje M. A. De Graaf, Frank A. Hindriks, Koen V. Hindriks

**Affiliations:** ^1^ Department of Information and Computing Sciences, Utrecht University, Utrecht, Netherlands; ^2^ Department of Ethics, Social and Political Philosophy, University of Groningen, Groningen, Netherlands; ^3^ Department of Computer Science, Vrije Universiteit Amsterdam, Amsterdam, Netherlands

**Keywords:** capacities, reasons, rights, robots, traits

## Abstract

The robot rights debate has thus far proceeded without any reliable data concerning the public opinion about robots and the rights they should have. We have administered an online survey (*n* = 439) that investigates layman’s attitudes toward granting particular rights to robots. Furthermore, we have asked them the reasons for their willingness to grant them those rights. Finally, we have administered general perceptions of robots regarding appearance, capacities, and traits. Results show that rights can be divided in sociopolitical and robot dimensions. Reasons can be distinguished along cognition and compassion dimensions. People generally have a positive view about robot interaction capacities. We found that people are more willing to grant basic robot rights such as access to energy and the right to update to robots than sociopolitical rights such as voting rights and the right to own property. Attitudes toward granting rights to robots depend on the cognitive and affective capacities people believe robots possess or will possess in the future. Our results suggest that the robot rights debate stands to benefit greatly from a common understanding of the capacity potentials of future robots.

## 1 Introduction

Human beings have inalienable rights that are specified in the Universal Declaration of Human Rights. But other entities can have rights too. Animals are commonly taken to have moral rights (Regan, 2004). And organizations have legal rights, including the right to own property and enter into contracts (Ciepley, 2013). But what about robots? Should they have rights? People spontaneously infer intentionality and mind when encountering robots which shows that people cognitively treat robots as social agents (de Graaf and Malle, 2019). But do robots have moral standing, as humans and animals do? Or do they merely have legal rights, just as organizations?

Agents can have moral standing as moral patients. For instance, animals are moral patients because they can suffer. More generally, a moral patient is an agent that can be wronged (Gunkel, 2012). If moral patients have rights, these serve to protect them from such wrongdoings. Agents can also have moral standing as moral agents. Human beings are moral persons, because they are rational and because certain things matter to them. Some of their rights allow or enable them to develop themselves or to live the kind of life they value. The debate about robot rights is commonly framed in terms of moral patiency (Gunkel, 2018). This suggests that they are meant to prevent others from wronging robots.

A third alternative has been proposed by Gunkel (2012), Gunkel (2018) and Coeckelbergh (2010), Coeckelbergh (2021), who defend a social-relational approach to robot rights. Moral patiency and personhood are properties of agents. According to the social-relational approach, the moral standing of robots depends instead on the social relations between humans and robots. Instead of being defined by its attributes, a robot’s moral status should be based on people’s social responses to robots (Gunkel, 2018), on how people relate to them, and on the value they have to humans (Coeckelbergh, 2021). In light of this, the social-relational approach can be regarded as human-centered. This is an interesting development particularly because robots cannot suffer and do not value things, which makes it problematic to grant them rights on the basis of their intrinsic properties.

The law treats organizations as legal persons. This notion of legal personhood is often said to be a legal fiction because organizations are not really persons. Because of this legal fiction, they can be granted legal rights. Such rights protect the interests of human beings. Robots might be granted legal rights for the same reason, but this would mean that we have to regard them as legal persons. However, the idea of legal robot rights also has met with controversy.

In 2016, the EU’s Committee on Legal Affairs suggested that “the most sophisticated autonomous robots” can have “the status of electronic persons with specific rights and obligations.” This committee requested a study on future civil law rules for robotics. This study was commissioned, supervised, and published by the “Policy Department for Citizens’ Rights and Constitutional Affairs,”^1^ resulting in a resolution by the Parliament.^2^ The study aimed to evaluate and analyze a number of future European civil law rules in robotics from a legal and ethical perspective. In an open letter, a coalition of politicians, AI/robotics researchers, industry leaders, health specialists, and law and ethics experts expressed concerns about this.^3^ They were worried in particular by the call on the EU commission to explore the implications of creating a specific legal status for robots to address issues related to, for example, any damage robots may cause.

At the same time, others have argued that we need to consider legal personhood for robots because current legal concepts of, for example, responsibility and product liability are no longer sufficient for ensuring justice and protecting those whose interests are at stake (Laukyte, 2019). Thus, robots challenge the law and legal institutions in new ways (Calo, 2015). This is vividly illustrated by the fact that a robot has already been granted citizenship rights (Wootson, 2017).

On the whole, there is little consensus on whether robots should have rights (see Darling (2016), Gunkel (2014), Levy (2009), Schwitzgebel and Garza (2015), Tavani (2018) for some proponents) or not (see Basl (2014), Bryson et al. (2017) for some opponents of this view). Others, such as Gerdes (2016) and Gunkel (2018), have argued that we should at least keep the possibility of granting rights to robots open. These conflicting views raise the question whether and how the debate can progress.

So far, the debate has involved mainly legal experts, philosophers, and policy makers. We, along with Wilkinson et al. (2011), believe that it will be useful to engage the public in the debate about robot rights. Rather than engaging in the debate ourselves, we have conducted an exploratory study investigating people’s attitudes toward robot rights through an online survey. To the best of our knowledge, this is the first study that explores layman’s opinions on granting robots rights. The main goals are *1*) to examine which reasons people find convincing for granting robot rights and *2*) how willing they are to grant such rights, while *3*) also administering people’s general perceptions of robots (appearance, mental capacity, and human-likeness) and *4*) investigating how these relate to their position on robot rights.

Our article is organized as follows. Section 2 justifies the design of the survey. It embeds it in the literature, it discusses contemporary psychological findings on people’s perceptions of robots, and it explains how the rights we consider relate to existing declarations of rights. Section 3 presents our research design and section 4 presents our findings. Section 5 discusses how these results relate to existing findings in HRI research, draws various conclusions, and points to future research directions.

## 2 Theoretical Background and Survey Design

Our work empirically investigates people’s attitudes toward the issue of granting robots rights by means of an online survey. This section introduces and substantiates the four main survey sections including items on the willingness to grant particular rights to robots in Section 2.1, how convincing several reasons are for granting robot rights in general in Section 2.2, the belief future robots may one day possess certain capacities and traits in Section 2.3, and a general image people have when picturing a robot in Section 2.4.

### 2.1 Rights

The main question that we are interested in here is what everyday people think about the kinds of rights (qualifying) robots deserve. We have broadly surveyed rights that have been granted or proposed for people (human beings), animals, corporations, and, more recently, specifically for robots. As we believe we should at least try to refrain from applying clearly biological categories to robots, we have rephrased our list of rights to match the (apparent) needs of robots, which inherently differ from biological entities (Jaynes, 2020). We have also tried to keep the formulation of rights concrete, simple, and short. As it is not possible to exhaustively determine what the needs (if any) of (future) robots will be, our list may not be complete even though we have tried to compile a list that is as comprehensive as possible. Table 1 lists the rights used in our study, where the *Source* column indicates the source from which we have derived a right. We refer to rights (and reasons below) by table and row number, for example, 1.1 refers to the right to make decisions for itself. This section discusses how we have translated existing rights to robot rights.

**TABLE 1:** List of robot rights used in the online survey.

Nr	Right	Source
	Should robots have the right to …	
1	make decisions for itself	ICESCR Art 1
2	select and block services that it provides	ICESCR Art 6
3	receive fair wages for the work they perform	ICESCR Art 7
4	access energy to recharge themselves	ICESCR Art 11
5	receive updates and maintenance	ICESCR Art 12
6	evolve and develop new capabilities over time	ICESCR Art 13
7	shape and form their own biography	ICCPR Art 6
8	not to be abused either physically or in any other way	ICCPR Art 7
9	be free to leave and return to any country, incl. its own	ICCPR Art 12
10	a fair trial	ICCPR Art 14
11	have freedom of expression through any media of their choice	ICCPR Art 19
12	collectively pursue and protect robot interests	ICCPR Art 22
13	vote for public officials	ICCPR Art 25
14	be elected for political positions	ICCPR Art 25
15	own property	UDHR Art 17
16	the pursuit of happiness	DAW Art 1
17	copy and duplicate themselves	DAW Art 5
18	not to be terminated indefinitely	DAW Art 6
19	enter into contracts	Ciepley, (2013)
20	store and process data they collect	Laukyte, (2019)

#### 2.1.1 Human Rights

Human rights have been documented in the Universal Declaration of Human Rights (UDHR).^4^ They have been laid down in two legally binding international agreements, the International Covenant on Civil and Political Rights (ICCPR)^5^ and the International Covenant on Economic, Social and Cultural Rights (ICESCR)^6^, both adopted in 1966. The rights that feature in these agreements are very different, particularly regarding their means of implementation.

The ICESCR contains economic, social, and cultural rights. These rights were considered to require a proactive role of the state involving financial and material resources. From the ICESCR, we derived rights 1.1-6. For 1.1, we changed “self-determination” into “make decisions for itself” to be more concrete. We assume that robots will be designed to provide specific services to humans (as per the origin of their name, cf., Oxford English Dictionary). As the right to work pertains to “the opportunity to gain his living by work he freely chooses,” we reformulated 1.2 in terms of the right to select or block services. As Chopra and White (2004) point out, the ability to control money is important in a legal system since “without this ability a legal system might be reluctant to impose liabilities” on robots; we, therefore, included 1.3. Since robots do not need food (they are artificial physical machines) but do need energy, we have 1.4. We translated “physical and mental health” into “updates and maintenance” (1.5) and “education” into “new capabilities” (1.6).

The ICCPR enumerates a number of civil and political rights or “classic freedom rights.” States enforce these rights primarily by not interfering with their citizens. In other words, they are to refrain from action in these fields. From the ICCPR we derived rights 1.7-14. To be suitable for our investigation, we had to adjust them in several respects. To avoid the strong biological connotations of life, we refer to forming a biography in 1.7, in line with Wellman (2018): “A life is a process that involves both goal-directed activities and projects that may succeed or fail and memories of what one has done in the past and what has befallen one […]. The concept of a life is a biographical not a biological concept.” We preferred “abuse” over “torture” in 1.8 though we recognize this does not cover “cruel punishment” which may be covered at least in part by 1.18. Right 1.10 was abbreviated to its core. Similarly, we included “freedom of expression” but only in part; we excluded references to (robot) “conscience” and “religion” in 1.11. Furthermore, we translated “freedom of association” and “trade unions” into the collective pursuit and protection of robot interests in 1.12. We split ICCPR Article 25 into two separate rights (as for robots they may have very different consequences, for example, in combination with 1.17). We chose to leave the mechanism of a “secret ballot” implicit. Finally, we derived 1.15 from the UDHR. We believe that most other articles from these declarations and covenants are covered (more or less) already by the rights that we have included or are (clearly) not applicable to robots.

#### 2.1.2 Animal Rights

Rights for nonhuman animals vary greatly by country. Some countries legally recognize nonhuman animal sentience. Others do not even have anti-cruelty laws. We derived three rights from The Declaration on Animal Rights (DAW)^7^ that were not yet covered by the rights discussed above. The declaration is still a draft and not yet a law, as most of the human rights are, though animal law exists and is continuously evolving in many countries.

Only the Declaration on Animal Rights refers explicitly to “the pursuit of happiness” as a right, which is why we included 1.16 as a separate item. To avoid the perhaps strong biological connotations with “reproduce” and “offspring”, we translated these into “copy and duplicate” in 1.17, which we believe is the more appropriate analogical terminology for robots. Similarly, we translated, for example, “slaughtered” and “killed” to “terminated indefinitely” in 1.18. We have added the qualification “indefinitely” to meet the objection of Jaynes (2020), who argues that “depriving power to the [robot] cannot be considered an act of murder, as the [robot]’s “personality” will resume once power has been restored to the system.” Finally, there might be a relation between this right and the right to life. After all, terminating a robot indefinitely would make shaping its own biography impossible. Even so, some argue that only those that have the potential for self-determination (ICCPR Article 1) and moral action (autonomy) can have a right to life. We regard the two as sufficiently distinct to include both.

#### 2.1.3 Corporate Rights

Corporations are created by means of a corporate charter, which is granted by the government. They receive their rights from their charter (Ciepley, 2013). As mentioned in the introduction, corporations are often seen as legal fictions. Chief Justice Marshall puts it in Dartmouth as follows: “A corporation is an artificial being, invisible, intangible, and existing only in contemplation of law. Being the mere creature of law, it possesses only those properties which the charter of its creation confers upon it” (Dartmouth College v. Woodward 1819, 636; our emphasis). Perhaps the most important right that corporations have is the right to enter into contracts (Ciepley, 2013). As it seems possible for robots to possess it, we include it as right 1.19.

#### 2.1.4 Robot-specific Rights

Finally, inspired by Laukyte (2019), we add right 1.20 to store and process data which arguably is associated specifically with robots.

### 2.2 Reasons for Granting Robots Rights

Many (combinations of) reasons have been put forward for granting robots rights. Miller (2015) maintains that robots “with capacity for human-level sentience, consciousness, and intelligence” should be considered entities that “warrant the same rights as those of biological humans.” Tavani (2018) thinks that a robot should have consciousness, intentionality, rationality, personhood, autonomy, and sentience to be eligible for rights. Strikingly, many of these properties are requirements for moral personhood. Laukyte (2019) states that the increasing autonomy, intelligence, perceptiveness, and empathy of robots shift our view away from robots as mere tools. These are among the main reasons for granting robots rights. Based on a review of the literature, we have tried to identify the main reasons that have been discussed so far (see Table 2).

**TABLE 2:** List of reasons used in the online survey.

Nr	Reason
	How convincing is it to grant robots rights when …
1	they can perceive the world around them
2	they can experience pain
3	they can experience pleasure
4	they can have feelings
5	when they can pay attention
6	when they have preferences
7	they can have memories
8	they can use language
9	they can independently make decisions and act on their own
10	they can take their own moral considerations into account
11	they have a conscience
12	they can make rational decisions
13	they are super-intelligent
14	human beings can no longer be held responsible for what robots do
15	they can learn
16	they appear humanlike
17	they can move around
18	they can understand others
19	they have a unique personality
20	they can love people
21	it is convenient to do so

#### 2.2.1 Consciousness

Consciousness is an important reason in the literature for granting robots rights. Levy (2009) claims that robots should be treated ethically by “virtue of their exhibiting consciousness.” It is common to distinguish between two kinds of consciousness, phenomenal consciousness on the one hand and access or functional consciousness on the other (Block, 1995; Torrance, 2012). Phenomenal consciousness requires sentience. As such, it is experiential and subjective. Think, for instance, of seeing, hearing, smelling, tasting, and feeling pain. Phenomenal conscious states encompass sensations, perceptions, feelings, and emotions. In contrast, access consciousness concerns awareness and plays an essential role in reasoning (Block, 1995). It is representational and makes mental content available for evaluation, choice behavior, verbal report, and storage in working memory (Colagrosso and Mozer, 2005).

Torrance (2012) states that “it is the phenomenal features of consciousness rather than the functional ones that matter ethically.” The main related reason that is often cited for granting entities moral status and rights is that they can suffer: they can experience pain from physical or emotional harm. The ability to (physically) suffer has also been one of the main reasons for granting rights to animals (Singer, 1974). We include the concrete reason items 2.1-5 for perception, suffering, experiencing pleasure, feelings, and attention. Note, however, that it is contested whether robots will ever be able to feel pain (see Levy (2009) contra versus Kuehn and Haddadin (2017) pro). We did not add a separate item for “consciousness.” Given how complex the notion is, this would not be meaningful.

Insofar as access consciousness is concerned, Freitas (1985) argues that “any self-aware robot that speaks [a language] and is able to recognize moral alternatives” should be considered a “robot person.” The EU draft report mentioned in the introduction also refers to the ability of robots to “make smart autonomous decisions or otherwise interact with third parties independently” to grant robots the status of an electronic personality. These items correspond to cognitive skills that humans have. We include reason items 2.6-9 for access-related phenomena. Although decision making involves preferences, we regard it as important to add it as a separate item.

#### 2.2.2 Autonomy

Another reason for assigning rights has been the ability to make decisions and perform actions independently, without any human intervention. This capability corresponds to the cognitive ability of humans to make decisions. It is not sufficient that a system can act without human intervention. That would be mere automation (the machine can act automatically) and does not capture the richer sense of what autonomy is. “To be autonomous, a system must have the capability to independently compose and select among different courses of action to accomplish goals based on its knowledge and understanding of the world, itself, and the situation.”^8^ Tessier (2017), moreover, adds that such decision making should be based on an understanding of the current situation.

Independent decision making and acting (without human intervention) is only one aspect of the notion of autonomy. Another reason for assigning rights is the ability to make decisions and to live your life according to your own moral convictions. Borenstein and Arkin (2016) also note that there is a difference in how the term “autonomy” is normally used in ethics in contrast with how it is used within AI: “the term ‘autonomy’ in the sense of how it is normally defined within the realm of ethics (i.e., having the meaningful ability to make choices about one’s life); within the realm of robotics, ‘autonomy’ typically refers to a robot or other intelligent system making a decision without a ‘human in the loop.’” The ability to distinguish right from wrong also has been put forward as an argument in favor of legal personhood (Chopra and White, 2004). This discussion motivated items 2.10-11.

#### 2.2.3 Rationality and Super-Intelligence

Rationality has been put forward as an important reason why humans have moral standing. According to Nadeau, “only machines can be fully rational; and if rationality is the basic requirement for moral decision making, then only a machine could ever be considered a legitimate moral agent. For Nadeau, the main issue is not whether and on what grounds machines might be admitted to the population of moral persons, but whether human beings qualify in the first place” (Gunkel (2012); see also Sullins (2010)). Solum (1992) argues that intelligence is a criterion for granting rights. Robots may become much smarter than the best human brains in practically every field. When robots outperform humans on every cognitive or intellectual task and become super-intelligent, some argue we should assign them robot rights. This discussion motivated items 2.12-13.

#### 2.2.4 Responsibility Gaps

In a communication to the members of the EU Parliament, before they voted on the Resolution on Civil Law Rules of Robotics on February 16, 2017, the intention to grant a legal status to robots was clarified as follows: “In the long run, determining responsibility in case of an accident will probably become increasingly complex as the most sophisticated autonomous and self-learning robots will be able to take decisions which cannot be traced back to a human agent.” Another argument that has been put forward is that if robots are able to perform tasks independently without human intervention, it will be increasingly difficult to point responsibility to a specific person or organization when something goes wrong (Danaher, 2016). Some scholars therefore propose that moral and legal responsibility should at some point be extended to robots (Wiener, 1954). This motivates reason 2.14. We added 2.15 because the ability of robots to learn has also been cited as a key reason for responsibility gaps, e.g., Matthias (2004).

#### 2.2.5 Humanlike Appearance and Embodiment

The fact that robots will at some point become indistinguishable from humans, both in their looks and the ways they behave, is for some scholars a reason to assign rights to robots. If robot appearance becomes very similar to that of human beings, one could argue that the basis for making a moral distinction between robots and humans is no longer tenable (Darling, 2016; Gunkel, 2018). This motivated item 2.16. Item 2.17 has been added to also emphasize the embodiment of robots and their physical ability of moving on their own capacity, as perhaps having the looks without being able to move will not do.

#### 2.2.6 Mind Perception, Personality, and Love

Understanding others’ minds (Gray et al., 2007; Gray et al., 2012) also seems relevant as Laukyte (2019) states that empathy of robots shifts our view away from robots as mere tools, and, moreover, this capacity matches with an item in the mental capacity scale (Malle, 2019). The notion of understanding others also raises the question about one’s own unique personality or identity and related notions of connectedness such as love as reasons for having rights, which motivated introducing items 2.18-20.

#### 2.2.7 Convenience

Finally, item 2.21 was added because one could also argue that from a more pragmatic stance, we should grant robots rights “simply” because they play a significant role in our society and granting robots rights may depend on “the actual social necessity in a certain legal and social order” (van den Hoven van Genderen, 2018).

### 2.3 Psychological Factors

People’s willingness to grant robot rights could result from their perceptions of future robots, and could be linked to the conceptions of moral patiency (and agency) presented in Section 1 by linking the philosophical interpretations of a robot’s moral standing to foundations in moral psychology research. Balancing on the intersection of philosophy and psychology, moral psychology research revolves around moral identity development and encompasses the study of moral judgment, moral reasoning, moral character, and many related subjects at the intersection of philosophy and psychology. Questions on how people perceive an entity’s moral status is often investigated with theories of mind perception.

Effects of human-likeness in human–robot interaction have been profoundly discussed (Fink, 2012; Złotowski et al., 2015). In our survey, we aimed to go beyond a robot’s anthropomorphic form to focus on the potential humanness of robots. A research body on humanness has revealed specific characteristics perceived as critical for the perception of others as human and distinguishes two senses of humanness (Haslam, 2006), which we included in our survey. First, *uniquely human* characteristics define the boundary that separates humans from the related category of animals and includes components of intelligence, intentionality, secondary emotions, and morality. Denying others such characteristics is called *animalistic dehumanization* in which others are perceived as coarse, uncultured, lacking self-control, and unintelligent, and their behaviors are seen as driven by motives, appetites, and instincts. Second, *human nature* characteristics define the boundary that separates humans from nonliving objects and includes components of primary emotions, sociability, and warmth. Denying others such characteristics is called *mechanistic dehumanization* in which others are perceived as inert, cold, and rigid, and their behavior is perceived as caused rather than propelled by personal will.

These two senses of humanness can also be linked to the perception of mind. According to Gray et al. (2007), the way people perceive mind in other human and nonhuman agents can be explained by two factors: agency and experience, where agency represents traits such as morality, memory, planning, and communication, and experience represents traits such as feeling fear, pleasure, and having desires. The agency dimension of mind perception corresponds to uniquely human characteristics, and the experience dimension links to human nature characteristics (Haslam et al., 2012). These two dimensions are linked to perceptions of morality such that entities high in experience and entities high in agency are considered to possess high moral agency (Gray et al., 2007) and thus deserving of (moral) rights.

However, perceiving mind, and consequently deserving of morality (Gray et al., 2007) and presumably rights, is regarded as a subtle process (de Graaf and Malle, 2019). In particular, the dual-dimensional space of mind perception has been challenged as several studies failed to replicate especially the agency dimension, e.g., Weisman et al. (2017). A recent series of studies provides consistent evidence that people perceive mind on three to five dimensions (i.e., positive and negative affect, moral and mental regulation, and reality interaction) depending on an individual’s attitude toward the agent (e.g., friend or foe) or the purpose of mind attribution (e.g., interaction or evaluation) (Malle, 2019), and our survey has therefore administered the mental capacity scale of Malle (2019).

In summary, previous HRI research shows that people’s ascription of humanness as well as mind capacity to robots affects how people perceive and respond to such systems. In line with the social-relational perspective to a robot’s moral standing (Gunkel, 2012; Gunkel, 2018; Coeckelbergh, 2010; Coeckelbergh, 2021), we will investigate how such perceptions of humanness and mind influence people’s willingness to granting rights to robots.

### 2.4 Appearance of Robots

Although what constitutes a robot can significantly vary between people (Billing et al., 2019), most people, by default, appear to have a humanlike visualization of a robot (De Graaf and Allouch, 2016; Phillips et al., 2017). Nevertheless, what appearance people have in mind is relevant for answering the question whether they are eligible for rights. It is not clear up front which kind of robots (if any) deserve rights (Tavani, 2018). Here, we only assume that robots are artificial (i.e., not natural, nonbiological) physically embodied machines. To get a basic idea of people’s perception of what a robot looks like, we include a simple picture-based robot scale (Malle and Thapa, 2017), Figure 1 in our survey.

**FIGURE 1:**
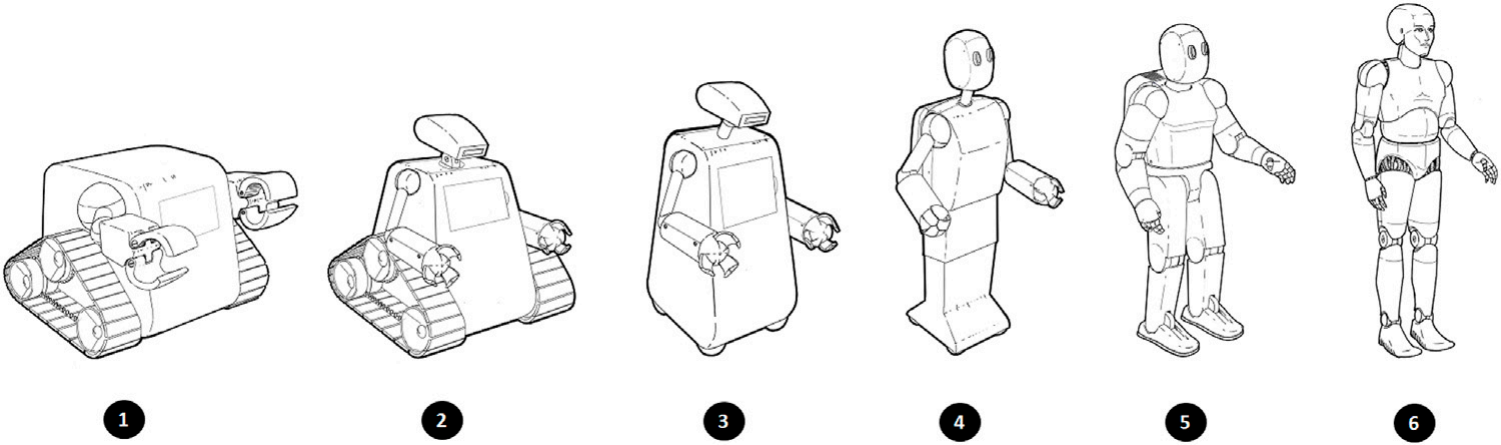
Robot appearance scale.

## 3 Methods

To examine layman’s opinions regarding robot rights, we have conducted an online survey administering participants’ willingness to grant particular rights to robots and their indication of how convincing several reasons are to grant those rights, while also administering people’s general perceptions of robots.

### 3.1 Procedure and Survey Design

After participants gave their consent, we introduced the survey topic describing that “[technological advancements], amongst other things, has initiated debates about giving robots some rights” and that “we would like to learn about [their] own opinions on several issues regarding the assignment of rights to robots.” The survey consisted of four randomly shown blocks (see Section 2) to avoid any order effects. The survey ended with questions regarding basic demographics, professional background, and knowledge and experience with robots. Average completion time of the survey was 11 (SD = 4:18) minutes, and participants’ contribution was compensated with $2.

The first block of the online survey contained one question asking participants which kind of robot appearance (see Figure 1) best resembles their image of a robot in general. The second and third block contained the reasons and rights items, respectively, of which the item selection was discussed in Section 2. The structure of each of the reason items was as follows and had the same format: “Suppose that robots [features]. How convincing do you think it is to grant rights to robots…*when [reason]*.” The [feature] slot is filled with capacities or features that robots will eventually possess to frame the question and put participants in a state of mind where they would presume these to be the case for (future) robots. The [reason] slot is filled with one of the 21 reasons from Table 2. For example, the item for the first reason is: “Suppose that robots can see, hear, smell, and taste. How convincing do you think it is to grant rights to robots…*when they can perceive the world around them*.” Participants were instructed to rate how appropriate they thought it would be to grant rights on a 7-points Likert scale. The format for the rights items is “Robots should have the right to [right]” where the [right] slot is filled with one of the rights from Table 1. For example, the item for the first right is: “Robots should have the right to…*make decisions for themselves.*” and participants were asked to rate how strongly they would oppose or favor granting the right on a 7-point Likert scale. The fourth block administered participants’ perceptions of future robots. To measure perceptions of capacities, we used the mental capacity scale developed by Malle (2019) consisting of the subscales affect (*α* = 0.94), cognition (*α* = 0.90), and reality interaction (*α* = 0.82). To measure perceptions of traits, we used the dehumanization scale developed by Haslam (2006) consisting of the subscales uniquely human (*α* = 0.85) and human nature (*α* = 0.98).

### 3.2 Participants

In April 2020, we initially recruited 200 USA-based participants from Amazon Mechanical Turk (De Graaf et al., 2021). In May 2021, we replicated our study by recruiting 172 EU-based participants from Amazon Mechanical Turk and 200 participants from Asia using Prolific. All participants, from either platform, had an approval rate of > 95%. For the EU and Asia samples, we administered a Cloze Test (Taylor, 1953) to ensure a good command in English, which led to the exclusion of 72 participants from Europe and 19 participants from Asia. In addition, 39 participants from the Asia sample were removed from further analysis because they had indicated growing up in Europe or the USA. The final data set used in our analyses included *n* = 439 participants (USA: *n* = 200, EU: *n* = 97, Asia: *n* = 142). In the EU sample, most participants were living in Italy (*n* = 36), Spain (*n* = 25) or Germany (*n* = 17). In the Asia sample, most participants were born and raised in China (*n* = 73), South Korea (*n* = 34), or Singapore (*n* = 17).

The complete sample included 53.3*%* men, 46.0*%* women, and 0.7*%* identified as gender-nonbinary. Participants’ age ranged from 20 to 71 (*M* = 35.5, SD  = 11.2), their educational level ranged from high school degree (23.2*%*) and associates degrees (11.4*%*) to bachelor’s, master’s, and doctoral degrees (65.1*%*), and 23.5*%* had a profession in computing and engineering. Most participants indicated having no or little knowledge about robots (52.1*%*) and never or rarely encounter robots in their daily life (71.9*%*), and participants mainly hold humanoid images of robots (61.3% selected picture five or six on the robot appearance scale). Measures on the robot appearance scale correlated only with the interaction capacity scale—and did so weakly (*r* = 0.181, *p* = 0.01)—and was therefore excluded from further analysis.

## 4 Results

### 4.1 Factor Analysis

As a first step, we conducted two separate factor analyses to reduce the individual items into a fewer number of underlying dimensions that characterize: *1*) the types of rights people are willing to assign to robots; and *2*) the types of reasons they consider for doing so. There were no outliers (i.e., Z-score of >3.29 ). Both sets of items were independently examined on several criteria for the factorability of a correlation. First, we observed that all 20 rights and all 21 reasons correlated at least 0.3 with at least one other right or reason, respectively, suggesting reasonable factorability. Second, the Kaiser-Meyer-Olkin measure of sampling adequacy was 0.97 for rights and 0.96 for reasons, well above the commonly recommended value of 0.6. Bartlett’s test of sphericity was significant in both sets, for rights (*χ* ^2^(190) = 6518.97, *p* < 0.001) and for reasons (*χ* ^2^(210) = 6822.39, *p* < 0.001), respectively. The diagonals of the anti-image correlation matrix were also all over 0.5. Finally, the communalities were all above 0.35, further confirming common variance between items. These overall indicators deemed factor analysis to be appropriate.

An eigenvalue Monte Carlo simulation (i.e., a parallel analysis) using the method described in (O’connor, 2000) indicated the existence of two and potentially three underlying dimensions for both the reasons and rights items. Solutions for both two and three factors were explored. We executed the factor analysis using an Alpha factors extraction (a method less sensitive to non-normality in the data (Zygmont and Smith, 2014)) with Oblimin rotations (allowing correlations among the factors)). A two-factor solution was preferred for both the reason and right items because of *1*) the leveling off of Eigenvalues on the screen plot after two factors; *2*) a low level of explained variance ( <4% ) of the third factor in both cases; and *3*) the lower number of cross-loading items.

The two reason factors had a total explained variance of 64.3*%*. Factor 1 revealed ten *cognition reasons* and factor 2 revealed nine *compassion reasons* both with strong factor loadings ( >.5 ; see Table 3 for the specific items). A total of two items were eliminated because they did not contribute to a simple factor structure and failed to meet a minimum criterion of having a primary factor loading of >.5 and/or had cross-loading of >.4 (i.e., having preferences, and making rational decisions). Internal consistency for each of the sub-scales was examined using Cronbach’s alpha, which were 0.93 for both cognition and compassion reasons. No increases in alpha for any of the scales could have been achieved by eliminating more items.

**TABLE 3:** Loading matrix of factor analysis on 21 reasons.

		Factor 1	Factor 2
Reasons		Cognition	Compassion
17	Moving around	0.926	−0.238
8	Using language	0.912	−0.112
5	Paying attention	0.852	−0.040
15	Learning	0.717	0.177
16	Appearing humanlike	0.653	0.038
7	Having memories	0.652	0.203
13	Super-intelligence	0.648	0.204
21	Convenience	0.616	−0.025
1	Perceiving the world	0.570	0.312
18	Understanding others	0.537	0.383
6	Having preferences	0.467	0.430
12	Making rational decisions	0.429	0.397
4	Having feelings	−0.186	0.967
11	Having a conscience	−0.146	0.907
10	Moral considerations	0.053	0.821
2	Experiencing pain	−0.057	0.821
20	Loving people	0.119	0.731
3	Experiencing pleasure	0.141	0.681
9	Acting on its own	0.171	0.659
14	Human responsibility impossible	0.128	0.542
19	Having a unique personality	0.377	0.502
	Eigenvalue	10.78	2.73
	% Explained variance	51.3	13.0
	Subscale Cronbach’s *α*	0.93	0.93

The two rights factors had a total explained variance of 64.1*%*. Factor 1 revealed thirteen *sociopolitical rights* and factor 2 revealed six *robot rights* both with strong factor loadings ( >.5 ; see Table 4 for specific items). One item was eliminated because it did not contribute to a simple factor structure and failed to meet a minimum criterion of having a primary factor loading of >.5 and/or had cross-loading of >.4 (i.e., pursuit of happiness). Internal consistency for each of the sub-scales was examined using Cronbach’s alpha, which were 0.95 for sociopolitical rights and 0.88 for robot rights, respectively. No increases in alpha for any of the scales could have been achieved by eliminating more items.

**TABLE 4:** Loading matrix of factor analysis on 20 rights.

		Factor 1	Factor 2
Nr	Right	Sociopolitical	Robot
13	Vote	0.985	−0.229
14	Be elected	0.936	−0.228
15	Own property	0.875	−0.020
17	Duplicate	0.642	−0.007
9	Cross nation borders	0.635	0.217
1	Self-decide	0.598	0.278
3	Fair wages	0.586	0.282
12	Pursue and protect interests	0.570	0.358
18	Not be terminated	0.564	0.250
19	Enter into contracts	0.561	0.261
7	Form own biography	0.560	0.290
2	Block services	0.531	0.276
11	Freedom of expression	0.519	0.389
16	Pursuit of happiness	0.474	0.456
5	Updates and maintenance	−0.138	0.871
4	Access to energy	−0.004	0.765
8	Not be abused	0.077	0.713
6	Self-development	0.209	0.605
10	A fair trial	0.357	0.549
20	Process collected data	0.151	0.504
	Eigenvalue	11.08	1.75
	% Explained variance	55.4	8.7
	Subscale Cronbach’s *α*	0.95	0.88

### 4.2 Cluster Analysis

As a second step, we explored the data using cluster analysis to classify different groups of people based on their opinions about rights for robots and reasons to grant those. A hierarchical agglomerate cluster analysis was performed using Ward’s method as a criterion for clustering (Ward, 1963; Murtagh and Legendre, 2011). Clusters were initially considered by visually analyzing the dendrogram (Bratchell, 1989) while considering the iteration history, significance of the F statistics, and the number of individuals in each cluster. This was done to ensure the cluster solution was stable, that there was a clear difference between clusters, and that each cluster was well represented (*n* > 15*%*).

The analysis resulted in three clearly distinguishable clusters. Chi-square tests revealed significant demographic differences between the clusters in terms of age (*χ* ^2^(4) = 10.78, *p* = 0.029) and continent (*χ* ^2^(3) = 25.54, *p* < 0.001), and marginally significant differences for educational level (*χ* ^2^(4) = 7.86, *p* = 0.097) and robot encounters (*χ* ^2^(2) = 5.28, *p* = 0.071). No significant differences were found for gender (*χ* ^2^(2) = 0.12, *p* = 0.941), profession (*χ* ^2^(2) = 0.22, *p* = 0.896), or robot knowledge (*χ* ^2^(2) = 3.97, *p* = 0.138). Participants in cluster 1 (*n* = 99) are more likely people from the US (*z* = 2.9) and possibly not aged 55 and older (*z* = −1.2), have a lower educational level (*z* = 1.9), and encounter robots occasionally or frequently (*z* = 1.8). Participants in cluster 2 (*n* = 245) are more likely people from Asia (*z* = 2.5) and possibly aged 30 and younger (*z* = 1.4), and possibly have a higher educational level (*z* = 2.1). Participants in cluster 3 (*n* = 93) are more likely people from Europe (*z* = 1.9) and aged 55 and older (*z* = 2.7), and possibly have never or rarely encountered robots (*z* = 1.9).

A series of one-way ANOVA tests showed significant differences between the three clusters in assessments of robot capabilities and traits as well as their opinions about rights for robots and reasons to grant those. Given a violation of the homogeneity of variance assumption and the unequal sample sizes between the three clusters, we have reported the Welch’s F-statistics (Tomarken and Serlin, 1986) (see Table 5). These combined results indicate that participants in cluster 1 seem to hold a *cognitive affective view* on robots being more positive toward granting robots rights, deeming the reasons for granting rights to be more convincing, and believing in higher potentials of future robot capacities and traits. Participants in cluster 2 seem to hold a *cognitive but open-minded view* on robots being more positive toward granting rights to robots as well as the cognitive and interaction capacities of robots, but being more skeptical toward the affective capacities of future robots while indicating compassion reasons to be convincing for granting robots rights. Participants in cluster 3 seem to hold a *mechanical view* on robots being only positive about future robots’ capacity for interaction but being rather negative toward granting rights, nor deeming the reasons for granting rights to be convincing, and being generally skeptical about the potentials of future robot capacities and traits.

**TABLE 5:** Average construct ratings for all participants and per cluster.

		All			Cluster 1		Cluster 2		Cluster 3			Welch’s ANOVA		
Construct		M	SD		M	SD	M	SD	M	SD		F(2,434)	p	Cohen’s d
Capacity														
Cognition		4.02	1.40		5.12	1.10	3.93	1.22	3.10	1.38		69.12	0.000	1.44
Affect		2.67	1.49		3.97	1.60	2.53	1.22	1.65	0.96		75.97	0.000	1.56
Interaction		5.77	1.26		6.35	0.79	5.75	1.14	5.23	1.67		24.87	0.000	0.88
Trait														
Human nature		3.43	1.26		4.53	1.12	3.33	1.03	2.50	1.07		83.01	0.000	2.53
Uniquely human		4.14	1.19		4.97	0.99	4.05	1.02	3.48	1.30		46.35	0.000	1.62
Reason														
Cognition		3.73	1.48		5.15	1.07	3.84	1.07	1.94	0.81		304.77	0.000	2.17
Compassion		4.62	1.74		5.96	0.62	4.90	0.81	2.47	1.16		340.12	0.000	2.37
Right														
Robot		5.02	1.37		6.21	0.58	5.19	0.84	3.32	1.46		1964.96	0.000	2.11
Sociopolitical		3.47	1.46		5.31	0.84	3.34	0.93	1.84	0.72		475.83	0.000	2.40

Tukey HSD significance are at p < 0.01 between all pairs.

### 4.3 Regression Analysis

Given our aim to uncover the minimum number of predictors which significantly explains the greatest amount of variance for both sociopolitical and robot rights, we ran a series of step-wise multiple regressions for each cluster separately.

#### 4.3.1 Explaining Sociopolitical Rights

For cluster 1 (*people with a cognitive affective view on robots*), the capacities, traits, and reasons to assign rights were significant predictors of participants’ readiness to grant robots sociopolitical rights (*F*(2, 96) = 14.36, *p* < 0.001). Together, the capacity of cognition (*β* = 0.420, *p* < 0.001) and cognition reason (*β* = − 0.188, *p* = 0.040) explained 23*%* of the variance. Readiness to grant sociopolitical rights was for cluster 1 participants associated with beliefs that robots will (eventually) possess cognitive capacities while considering cognition reasons had a negative effect on their readiness to grant sociopolitical rights. For cluster 2 (*people with a cognitive but open-minded view on robots*), the capacities, traits, and reasons to assign rights were significant predictors of participants’ readiness to grant robots sociopolitical rights (*F*(1, 243) = 57.29, *p* < 0.001). The capacity of affect (*β* = 0.437, *p* < 0.001) was the sole predictor explaining 19% of the variance. Readiness to grant robots sociopolitical rights was for cluster 2 participants associated with beliefs that robots will (eventually) possess affective capacities. For cluster 3 (*people with a mechanical view on robots*), the capacities, traits, and reasons to assign rights were significant predictors of participants’ readiness to grant robots sociopolitical rights (*F*(3, 87) = 21.94, *p* < 0.001). Together, the capacity of cognition (*β* = 0.537, *p* < 0.001), the trait of uniquely human (*β* = − 0.246, *p* = 0.028), and cognition reason (*β* = 0.421, *p* < 0.001) explained 41% of the variance. Readiness to grant robots sociopolitical rights was for cluster 3 participants associated with beliefs that robots will (eventually) possess cognition capacities but lacking traits of intelligence, intentionality, secondary emotions, and morality (uniquely human) while considering cognition reasons positively affected their readiness to grant sociopolitical rights.

#### 4.3.2 Explaining Robot Rights

For cluster 1 (*people with a cognitive affective view on robots*), the capacities, traits, and reasons to assign rights were significant predictors of participants’ readiness to grant robots rights (*F*(1, 97) = 15.09, *p* < 0.001). The capacity of interaction (*β* = 0.367, *p* < 0.001) was the sole predictor explaining 14*%* of the variance. So, for cluster 1 participants, their belief that robots will (eventually) possess interaction capacities seems to be enough to grant the rights in our robot rights dimension to robots. For cluster 2 (*people with a cognitive but open-minded view on robots*), the capacities, traits, and reasons to assign rights were significant predictors of participants’ readiness to grant robots the rights in our robot rights dimension (*F*(3, 241) = 17.26, *p* < 0.001). Together, the capacity of interaction (*β* = 0.278, *p* < 0.001), the trait of human nature (*β* = 0.151, *p* = 0.013), and compassion reason (*β* = 0.200, *p* = 0.001) explained 17% of the variance. So, for cluster 2 participants, besides (eventually) possessing interaction capacities, robots will (eventually) have the traits of primary emotions, sociability, and warmth (human nature) to grant robot rights while considering compassion reasons further positively affected their readiness to do so. For cluster 3 (*people with a mechanical view on robots*), the capacities, traits, and reasons to assign rights were significant predictors of participants’ readiness to grant robots the rights in the robot rights dimension (*F*(3, 87) = 11.14, *p* < 0.001). Together, the capacity of cognition (*β* = 0.304, *p* = 0.002) as well as cognition (*β* = 0.209, *p* = 0.045) and compassion (*β* = 0.222, *p* = 0.028) reasons explained 25% of the variance. So, for cluster 3 participants, their readiness to assign the rights in the robot rights dimension to robots was justified by their beliefs that robots will (eventually) possess cognitive capacities while considering both cognition and compassion reasons positively affected their readiness to do so.

## 5 Discussion

Current discussion on robot rights is dominated by legal experts, philosophers, and policy makers. To consider the opinion of lay persons in the policy debate, in line with the social-relational perspective to a robot’s moral standing (Gunkel, 2012, 2018; Coeckelbergh, 2010, 2021), we explored people’s attitudes toward the issue of granting rights to robots in an online survey. A factor analysis has again identified two main dimensions for both reasons and rights, replicating our previous findings with the US-only sample (De Graaf et al., 2021). The reason dimensions consist, on the one hand, of mainly *cognition reasons* (e.g., moving around, language, attention, learning) with only two other at face value unrelated items (i.e, humanlike appearance and convenience) as reasons for granting robots rights, and affect-related *compassion reasons* (e.g, feelings, conscience, pain, moral considerations) on the other hand with only one at face value unrelated item (i.e., acting on one’s own). It thus appears that people’s perspective on robot affect and cognition plays an important role in the context of granting robots rights, which is also in line with the results of our cluster and regression analysis.

The first rights dimension, labeled *sociopolitical rights*, consists mainly of items associated with the freedom to do what one wants (e.g., vote, duplicate, cross borders, self-decide, shape one’s biography) and to be treated fairly (e.g., be eligible for election, own property, fair wages). A clearly different second dimension, labeled *robot rights*, mainly consists of items associated with a robot’s technical needs to function properly (updates, energy, self-development, process data) and the item to not be abused. One explanation why this last item is also associated with this dimension is that the right to not be abused was perceived as damaging other people’s property. These two rights dimensions reveal that people tend to differentiate between more general sociopolitical rights and those associated with a robot’s functional needs.

The average ratings for the various scales used in our study show that only the capacity of reality interaction (e.g., learning, verbally communicating, moving, perceiving the world) had high overall agreement that robots can do this well (see Table 5). People, thus, generally tend to have a rather positive view on the capabilities of (future) robots regarding their ability to (socially) interact with their environment, irrespective of their user characteristics (e.g., age, gender, continent, robot experience). The interaction capacity also predicts readiness to grant robot rights. The high averages on this scale indicate a high willingness to grant robot rights to robots (except for *people from EU, those aged 55 and older, and those less familiar with robots*, who tend to be more skeptical). Most people (about 80*%*) thus agree that robots should be updated, have access to energy, process collected data, and not be abused.

This is different for sociopolitical rights (e.g, voting, fair wages, and the right not to be terminated) which *people from cluster 1* (i.e., those who are most likely from the US, and possibly not aged 55 and older, have a lower educational level, and have encountered robots occasionally or frequently) seem to be most willing to grant to robots. This may be explained by our finding that these people are also more optimistic about the possibility that future robots can have affect, cognition, and human traits. Moreover, there is a strict order where people from cluster 1 are significantly more willing to grant sociopolitical rights than people from cluster 2 (i.e., those who are more likely from Asia, and possibly aged 30 and younger and have a higher educational level) followed by people from cluster 3 (i.e., those who are most likely from Europe and aged 55 and older, and possibly have never or rarely encountered robots) being least willing to do so.

Our findings suggest that it is more likely that people from the US are very optimistic about the potential of robots in general and are more likely to assign them rights, people from Asia are positioned somewhere in the middle on these issues, and people from Europe are overall much more skeptical. Our findings are somewhat similar to those of Bartneck et al. (2007) who also find that people from the US are the most positive, more so than Japanese, who appear in turn more positive than Europeans. Although one might be tempted to conclude there is a cultural link between assigning rights to robots from this, more evidence is needed to conclude such a relation. Note that our continent-based samples do *not* match with clusters (sizes differ with the US sample a size of *N* = 200 vs. cluster 1 a size of *N* = 99, the Asia sample a size of *N* = 142 vs. cluster 2 with a size of *N* = 245, and the EU sample with a size of *N* = 97 vs. cluster 3 with a size of *N* = 93). MacDorman et al. (2008) also do not find any evidence for strong cultural differences between the US and Japan. A cultural interpretation of our findings therefore seems premature and would require more research to support such conclusions.

Based on our cluster analysis, we can conclude that *people from cluster 3* (i.e., those who are more likely from Europe and aged 55 and older, and possibly have never or rarely encountered robots) generally have a more *mechanical view* of robots and are more skeptical about robots having cognitive or affective capacities or humanness traits. This is in line with a tendency for mechanistic dehumanization in this group. Because cognition and affect-related reasons are a predictor for this group, only if these capacities will be realized are they willing to grant sociopolitical rights. *People from cluster 2* (i.e., those more likely from Asia, possibly aged 30 and younger, and possibly with a higher education level) have a significantly more positive view and believe robots will have cognitive capacities and human traits, but they are less inclined to believe that robots will have affects, which for them is important to grant sociopolitical rights. This group appears to have a *cognitive view* of robots but is more skeptical about affective capacities. Note that all groups more strongly believe that robots will have cognitive rather than affective capacities (*see* Table 5). In contrast, *people from cluster 3* (i.e., those more likely from the US, and possibly not aged 55 and older, have a lower education level, and have encountered robots occasionally or frequently) have a very positive view on all capacities and traits of future robots. It appears that they have a *cognitive-affective view* of robots.

In our analysis, we did not find many strong relations between demographical factors and people’s views on assigning them rights (with the exception of age and continent), which is in line with the findings reported in MacDorman et al. (2008) which also does not find such relations. Flandorfer (2012) has reported on a link between age, experience, and attitude toward robots. In this work, it appears a younger age is associated with higher exposure to and more positive views on new technology in general, but we did not find such a trend. Finally, our findings overall are similar to those reported in our previous work (De Graaf et al., 2021) which only analyzed the US sample. One noticeable difference is that in our current analysis, we found only three instead of four clusters which are correlated with the continents associated with the three samples we collected. The fact we had four groups in our previous work is explained by the differences in experience with robots that does not play a differentiating role in our current analysis.

### 5.1 Limitations and Future Work

As any study, ours has some limitations. First, the three samples from the US, EU, and Asia varied significantly in division of age category and educational level. Regarding age, the US sample had an overrepresentation of people aged 50 and over, and the Asia sample had an overrepresentation of people aged 30 and younger. These demographics are actually quite similar to the actual population demographics in these continents.^9^ Regarding educational level, the US sample had an overrepresentation of people with a high school degree, and the Asia sample had an overrepresentation of people with a bachelor’s, master’s, or doctoral degrees.

Second, participants may have interpreted the survey items differently, particularly the reason items because of their conditional nature. We asked to suppose robots had certain capabilities or features and assess their willingness to grant rights *if* that were the case. Similarly, for the robot rights, which may have been granted more easily because participants read those more as operational requirements for robots rather than as rights. Future work should address any potential difficulty with interpreting these conditionals (Skovgaard-Olsen et al., 2016) to further validate our items and underlying dimensions regarding rights and reasons to grant them. A potentially interesting approach for such future work would be to relate our findings to the more general literature on technology acceptance (e.g., to understand how experience with robots factors into attitudes of people (Turja and Oksanen, 2019)) or to compare the current reasons to grant robots rights and the mental capacities (Malle, 2019) revealing potential missing coverage in the reasons. Finally, future research should explore the effect of a robot’s physical appearance on granting robots rights beyond the mechanical-humanoid dimension applied in our study.

### 5.2 Conclusion

Our study presents a survey design to empirically investigate the public opinion about robot rights. There appears to be an overall consensus about the interactive potential of robots. We found that people are more willing to grant basic robot rights such as access to energy and the right to update to robots than sociopolitical rights such as voting rights and the right to own property. We did not find any strong relation between demographic factors such as age or other factors such as experience with robots or of geographical region with the willingness to assign rights to robots. We did find, however, that beliefs about the (future) capacities of robots influence this willingness. Our results suggest that, in order to reach a broad consensus about assigning rights to robots, we will first need to reach an agreement in the public domain about whether robots will ever develop cognitive and affective capacities.

## Data Availability

The raw data supporting the conclusions of this article will be made available by the authors, without undue reservation.

## Footnotes

1 Draft Report with Recommendations to the Commission on Civil Law Rules on Robotics, Committee on Legal Affairs, https://www.europarl.europa.eu/doceo/document/JURI-PR-582443_EN.pdf?redirect, accessed February 23, 2020.

2 European Parliament resolution of 16 February 2017 with recommendations to the Commission on Civil Law Rules on Robotics (2015/2103(INL), https://eur-lex.europa.eu/legal-content/EN/TXT/?uri=CELEX%3A52017IP0051, accessed October 5, 2020.

3 Open Letter to the European Commission Artificial Intelligence and Robotics, http://www.robotics-openletter.eu/, accessed August 13, 2020.

4 Universal Declaration of Human Rights, https://www.un.org/en/universal-declaration-human-rights/, accessed on March 1, 2020, which was adopted in 1948 by the United Nations General Assembly.

5
https://www.ohchr.org/en/professionalinterest/pages/ccpr.aspx, accessed March 1, 2020.

6
https://www.ohchr.org/en/professionalinterest/pages/cescr.aspx, accessed March 1, 2020.

7
https://declarationofar.org/, accessed March 1, 2020.

8 Defense Science Board Summer study on autonomy, United States Defense Science Board, https://www.hsdl.org/?view&did=794 641, accessed March 13, 2020.

9 2019 Revision of World Population Prospects, United Nations, https://population.un.org/, accessed on September 3, 2021.
